# Assessment of the quality of the vital registration system for under-5 mortality in Yucatan, Mexico

**DOI:** 10.1186/s12963-022-00284-5

**Published:** 2022-02-08

**Authors:** Bernardo Hernandez, Elsa Rodriguez Angulo, Louisa M. Johnson, Erin B. Palmisano, Ricardo Ojeda, Rafael Ojeda, Salvador Gómez Carro, Alan Chen, Joseph Camarda, Casey Johanns, Abraham Flaxman

**Affiliations:** 1 Institute for Health Metrics and Evaluation (IHME), 3980 15th Ave NE, Seattle, WA 98195 USA; 2 Universidad Autónoma de Yucatán (UADY), Mérida, Mexico; 3 Secretaría de Salud de Yucatan, Mérida, Mexico

**Keywords:** Child mortality, Neonatal mortality, Mexico, Vital registration

## Abstract

**Introduction:**

Vital registration is an important element in health information systems which can inform policy and strengthen health systems. Mexico has a well-functioning vital registration system; however, there is still room for improvement, especially for deaths of children under 5. This study assesses the quality of the vital registration system in capturing deaths and evaluates the quality of cause of death certification in under-5 deaths in Yucatan, Mexico.

**Methods:**

We collected information on under-5 deaths that occurred in 2015 and 2016 in Yucatan, Mexico. We calculated the Vital Statistics Performance Index (VSPI) to have a general assessment of the vital registration performance. We examined the agreement between vital registration records and medical records at the individual and population levels using the chance-corrected concordance (CCC) and cause-specific mortality fraction (CSMF) accuracy as quality metrics.

**Results:**

We identified 966 records from the vital registry for all under-5 deaths, and 390 were linked to medical records of deaths occurring at public hospitals. The Yucatan vital registration system captured 94.8% of the expected under-5 deaths, with an overall VSPI score of 87.2%. Concordance between underlying cause of death listed in the vital registry and the cause determined by the medical record review varied substantially across causes, with a mean overall chance-corrected concordance across causes of 6.9% for neonates and 46.9% for children. Children had the highest concordance for digestive diseases, and neonates had the highest concordance for meningitis/sepsis. At the population level, the CSMF accuracy for identifying the underlying cause listed was 35.3% for neonates and 67.7% for children.

**Conclusions:**

Although the vital registration system has overall good performance, there are still problems in information about causes of death for children under 5 that are related mostly to certification of the causes of death. The accuracy of information can vary substantially across age groups and causes, with causes reported for neonates being generally less reliable than those for older children. Results highlight the need to implement strategies to improve the certification of causes of death in this population.

**Supplementary Information:**

The online version contains supplementary material available at 10.1186/s12963-022-00284-5.

## Introduction

Vital registration (VR) is the system in which governments record the number of births and deaths, including causes of death. It is recognized as a crucial element in a health information system, and well-functioning VR is associated with better health outcomes and directly benefits individuals and policy [1]. Countries and international organizations have made important efforts to strengthen VR systems to inform proper planning and evaluation of health systems, especially in developing countries [1]. However, progress to improve these systems has been modest, with the percentage of deaths registered globally increasing from 36% in 2000 to 38% in 2012 [2].

Mexico has conducted various activities to improve its VR and is considered a country with a well-functioning VR system based on its quality and completeness since the 2000s [3, 4]. The Mexican VR system is managed by the National Institute of Statistics (INEGI) in conjunction with the Secretary of Health and the Civil Registry offices. Additionally, the Center for Disease Classification (CEMECE) monitors the quality of coding of causes of death. At the national level, INEGI has established an automated coding system for the classification of underlying cause of death, the Automated Coding of Medical Entities (ACME) system, which carries out a substantial proportion of coding [5, 6], although the proportion of cases coded with this system varies by State.

In a study conducted in 2014 that evaluates VR systems using a composite index derived from different dimensions, Mexico’s VR system is ranked 29th out of 129 countries, being considered as “very high quality” [7]. However, there still exists room for improvement. The Mexican VR system relies on information regarding causes of death registered in death certificates, with most of certification completed by physicians [8]. The process to generate mortality estimates is complex, and even with good supervision of the coding of causes of death, errors can be introduced into the data if causes of death are not correctly stated in the death certificate. Another potential source of error is the use of what are known as “garbage codes,” which are ill-defined codes that do not offer a definite underlying cause of death. The use of these garbage codes can vary according to the place where the death occurs and the personnel certifying the cause of death [9].

In addition to comparing VR to death certificates, there is a substantial body of literature assessing the validity of death certification by comparing the underlying cause of death in the death certificate with medical records. These studies have high heterogeneity in their methodology and overall show a wide variation in the concordance between death certificates and medical records [10]. Previous research has found better concordance between death certificates and medical records for adults than for children/neonates [11, 12] [13]. Studies in Mexico also suggest that, despite having a VR system in place, problems in the certification process can affect the quality of cause of death certification, especially in the case of children where the overall concordance is low [11, 13].

The State of Yucatan, located in the southeast of Mexico, shares the structure and functioning of the Mexico VR system described above. The epidemiologic profile of the state is similar to the national profile, except for a higher prevalence of infectious diseases among children under 5 [14]. Specialized health care for children is concentrated in a small number of hospitals located in the cities of Merida, the capital of the state, and in Valladolid. Despite having a VR system in place with ongoing activities to improve it, there has been no systematic assessment of its quality. In Yucatan, studies have been done regarding filling out death certificates in cases of maternal deaths, but no studies have been done to assess the quality of registration for children [8, 15]. Therefore, the objective of this study is to assess the overall performance of the VR system for deaths of children under the age of 5 in the state of Yucatan by evaluating different dimensions of the VR system and by calculating the concordance between cause of death information obtained from medical death certificates relative to a rigorously defined gold-standard diagnosis based on medical records in health facilities from the Ministry of Health in Yucatan for deaths that occurred during 2015–2016.

## Methods

In order to assess the quality of the VR system, our team compiled data on all deaths of children under the age of 5 that occurred in the state of Yucatan during 2015–2016. We first identified all relevant records from the VR system. Second, we pulled the medical records from hospitals for all patients that were identified through the VR system. Lastly, a team of trained doctors reviewed all medical records and re-coded causes of death if the record contained incomplete or vague information.

### Data collection

The data collection team obtained 966 VR records from the Ministry of Health of Yucatan for all deaths of children under the age of 5 years which occurred in the state of Yucatan, Mexico, during 2015 and 2016. These records do not include deaths due to injuries, which were not released to our team. Deaths due to injuries accounted for about 6.4% of all deaths in the state of Yucatan, Mexico, during 2015–2016 [16].

### Medical record review

The data collection team searched for medical records for the deaths identified in the VR from all seven public hospitals of the Ministry of Health of Yucatan.

Part of the medical record review included classifying records by level of quality as well as identifying causes of death based on information from the medical record. To identify the cause of death, we use the gold-standard criteria developed as part of the Population Health Metrics Research Consortium (PHMRC) verbal autopsy study [17]. These criteria define the laboratory tests, imaging results, and recorded clinical signs of illness required to properly diagnose each underlying cause of death. Medical records were classified into three quality categories according to their information on causes of death: Level 1 is classified as medical records that contained all the appropriate information for each cause and represented cases where there was the highest certainty in the correctness of the final diagnosis. Records with only some of the required tests to assign a cause of death or with tests that are less definitive were classified as Level 2 or 3, respectively. Records that did not contain enough documented clinical evidence were classified as “undetermined.” The criteria for each cause and level are listed in Additional file 1.

A team of five doctors underwent extensive training in medical record review and extraction, including computer-based data extraction of medical records using an electronic form, which was programmed to include the diagnostic criteria for each cause and verify the assigned gold-standard level. The electronic data extraction form verified the consistency of the information collected from the medical records and double-checked the validity of the gold-standard level assigned.

All records from the VR were linked to medical records using the full name of the decedent as well as other information including birthday and national unique population registry number. This ensured that linked records matched with as much certainty as possible. While reviewing medical records, we found 41 additional records of hospital deaths that could not be linked to the VR system and collected information from these records as well.

Ethical approval for the study was granted by the Internal Review Boards of the University of Yucatan, the Hospital Agustín O’Horán (Merida, Yucatan), and the University of Washington. To protect confidentiality of the information, at the time of analysis, a study ID was assigned to each case and all identifying information was removed from the data.

### Recoding

Coding of causes of death in the State of Yucatan is done mostly manually, and automated coding is using for verification of some cases only. Therefore, there is still the possibility of errors in the coding of causes of death that may affect the accuracy of mortality statistics. In order to explore this possibility, a team of three trained cause-of-death coders independently reviewed the death certificates for all records found in the VR and assigned the appropriate ICD-10 codes at the three digit level for each death according to the information on causes of death available in the death certificate. Cases where the coders disagreed were re-reviewed, and a final decision was made about the underlying cause of death. This resulted in a second vital registry database with recoded underlying ICD-10 codes.

## Analysis

After compiling the data, we calculated several metrics to assess both the overall quality of the VR system as well as the individual and population levels of agreement between the VR records and the medical records. We first calculated a metric called the Vital Statistics Performance Index (VSPI), which measures five dimensions of the VR system and allows us to compare the Yucatan VR system with other systems around the world. We then measured the level of agreement between VR and medical records by calculating the chance-corrected concordance (CCC) for the individual level and the cause-specific mortality fraction (CSMF) accuracy for the population level.

### Vital statistics performance index

We first calculated the Vital Statistics Performance Index (VSPI) for the subset of the Yucatan VR pertaining to deaths of children under the age of 5 years, following the methodology proposed by Phillips et al. [7]. The VSPI is a standardized metric for assessing VR systems and provides useful information for benchmarking and tracking improvement in health information systems over time [1]. The VSPI consists of five dimensions: (1) the quality of cause-of-death reporting measured by the proportion of garbage coding, (2) the quality of age and sex reporting measured by the proportion of records with unspecified age or sex, (3) internal consistency measured by the proportion of records with a cause that is medically impossible given the reported demographics, (4) completeness of the death reporting compared to estimates from demographic methods, and (5) the level of cause-specific detail measured by the number of distinct causes. A sixth dimension involving the timeliness of reporting can also be included. Since our study only focuses on two recent years, we excluded this dimension. High-quality VR data are defined as having greater than 90% completeness and less than 10% of garbage codes, appearing on the registrations. Low-quality data have less than 70% completeness or have garbage codes appearing on greater than 20% of registrations [4].

To determine the expected number of deaths for the completeness indicator, we extracted total mortality estimates from the Global Burden of Disease (GBD) 2016 study [14]. The length of the cause list used to classify deaths is usually calibrated to causes of death for all age groups aggregated to a cause list suitable for policy purposes. Our study contains a small sample and focuses on deaths in the under-5 population, so we were not able to compare the number of reported causes to expected total used in the original study. However, all the deaths in the registry are coded with detailed ICD-10 codes, so we assumed the cause-specific detail was adequate to warrant no penalty and that unreported causes represent causes of death that are not present in this population. Therefore, the VSPI is the product of the four remaining categories. We calculated the other indicators using the information from the VR records. After calculating the raw score on each dimension, we transformed these scores using accuracy weights, as proposed in the original simulation study [7]. The overall VSPI score is a product of the dimensions of the index and indicates the percentage of cases meeting all the dimensions. We compared the overall VSPI score calculated only using the dimensions of garbage codes, age/sex unspecified, impossible diagnoses, and completeness vs. the same score calculated using data from the Global Burden of Disease Study for Mexico 2013–14 and Yucatan 2015–2016, which was recalculated using only these categories to maintain comparability.

### Concordance between VR and medical records

We assessed the quality of the cause of death information listed in the VR by comparing it to the cause determined by the medical record review. The records in the VR contain an underlying cause of death as well as secondary ICD-10 codes for the sequence of causes that led to the underlying cause of death. To estimate the quality of the information at the individual level, we compared, for each case, the cause of death stated in the VR versus the one derived from the medical record. We conducted the concordance analysis first with only the listed underlying cause. We then reanalyzed the data, examining all listed causes in the VR for each record. If any listed cause matched the cause derived from the medical review, we considered the case concordant. We also examined concordance using the recoded ICD-10 codes which resulted from our review. To take into account that a case can be concordant just by chance, we calculated chance-corrected concordance (CCC) to determine the degree of similarity for each record [18].

We then assessed the quality of the cause-of-death information at the population level by comparing the cause-specific mortality fraction (CSMF) accuracy derived from the information in the VR and the medical records, estimating the CSMF accuracy to determine the reliability of population-level estimates generated from VR [19].

CSMF accuracy is affected by the cause composition of the sample. To provide a more robust estimate of the population-level accuracy, we resampled the data 500 times to calculate CSMF accuracy. For each resampled dataset, we first drew a cause distribution from an uninformative Dirichlet distribution and then sampled observations to give this cause. We calculated the median CSMF accuracy across the 500 resampled datasets as the population-level accuracy. We also fit a linear regression for the cause fraction of each cause across the 500 resampled datasets [12]. If the listed cause matched the true cause, the slope of the fit line would be 1 with an intercept of 0. Deviation from this indicates over- or under-estimation of the number of cases due to a given cause of death in the sample. Lastly, we calculated root mean squared error (RMSE) for the linear fit, which measures the precision of the estimate, where lower values indicate greater correlation between the CSMF calculated from the VR and our gold standard.

## Results

### Vital registry

The official state of Yucatan VR contained a total of 966 under-5 deaths during 2015–2016, giving a mortality rate of 13.4 deaths per 1000 live births, which is lower than the Mexican average of 16 deaths per 1000 live births during the same time period [20]. Table 1A displays the population characteristics for records in the VR. Across both years, there were a total of f 438 (45.3%) neonatal deaths (occurring within 28 days of birth) with the remaining 528 (54.7%) being children between the ages of 28 days and 5 years. 812 (84.1%) deaths in the VR were recorded at a hospital, and 431 (44.6%) of these listed a hospital administered by the Ministry of Health. Of the 431 deaths occurring at hospitals administered by the Ministry of Health, we were able to find and match complete medical records detailing the diagnosis, treatment and evolution of the case for 334 (77.5%) of the deaths, of which 318 (73.8%) occurred at Hospital General Agustín O’Horán, the primary government hospital in the capital city of Merida.

**Table 1:** Description of the (A) vital registry (VR) dataset (N = 966). (B) medical record review (MRR) dataset (N = 390)

Measure	*N*	Percent (%)
**(A)**		
Year		
2015	506	52.4
2016	460	47.6
Sex		
Female	415	43.0
Male	539	55.8
Unspecified	12	1.2
Age group		
Neonate	438	45.3
Child	528	54.7
Hospital listed		
Ministry of Health hospital	431	44.6
Non-Ministry of Health hospital	381	39.5
No hospital listed	154	15.9
**(B)**		
Age group		
Neonates	210	53.8
Child	180	46.2
Diagnostic criteria		
Level 1	237	60.8
Level 2	35	9.0
Level 3	43	11.0
Undetermined	75	19.2

### Medical record review

The team was able to extract medical records for 390 (90.5%) out of the 431 total cases from the Ministry of Health hospitals, as shown in Table 1B. In total, 210 (53.8%) records were neonatal and 180 (46.2%) were child records. The team of doctors evaluated each record and assigned categories to reflect the certainty in the assignment of cause of death, as defined by the PHMRC-defined gold-standard criteria, with Level 1 being the highest certainty and Level 3 being the lowest certainty. Of the records, 237 (60.8%) met the Level 1 diagnostic criteria, 35 (9.0%) met the Level 2 criteria, 43 (11.0%) met the Level 3 criteria, and the remaining 75 (19.2%) did not contain enough evidence to meet any of the predefined criteria. The team was unable to link 56 medical records to records in the VR.

### Vital statistics performance index (VSPI)

For all under-5 deaths appearing in the VR (*N* = 966), 122 (12.6%) listed garbage code, 34 (3.5%) listed an underlying cause of death that is deemed biologically impossible, and 12 (1.2%) were missing either age or sex. Compared with mortality estimates from the GBD project, the Yucatan vital registry captured 94.8% of the under-5 deaths. After adjusting the raw indicator values by the accuracy weights described in the Phillips et al. study, the product of the transformed values gave an overall VSPI score of 87.2% [7].

We then recalculated indicators that depended on causes of death using the recoded ICD-10 codes. After this recalculation, 112 deaths (11.6%) still listed as underlying cause of death garbage code, 25 (2.6%) listed an impossible ICD-10 code, and 12 (1.2%) were still missing either age or sex. Compared with mortality estimates from the GBD project, the data were 94.8% complete. Therefore, only the cases that included as underlying cause of death a cause deemed impossible or ill-defined were affected by the re-coding. After transformation, this gave a final adjusted index score of 88.5%.

Table 2 compares the VSPI of the Yucatan VR system for both original and recoded values to previously published estimates for Mexico (all age groups) as well as the state of Yucatan (children under the age of 5). Values for garbage codes, unspecified age or sex, and medically impossible diagnoses are all subtracted from 100% so that higher values are preferable to lower, to be consistent with the other indicators.

**Table 2:** Vital Statistics Performance Index (VSPI) scores for under-5 deaths in Yucatan, Mexico, 2015–2016

Dimension	Original ICD codes		Recoded ICD codes		GBD estimates	
	Raw (%)	Weighted (%)	Raw (%)	Weighted (%)	Mexico^a^ (%)	Yucatan^b^ (%)
Garbage codes^c^	87.4	91.8	88.4	92.4	92.8	92.1
Age/sex unspecified^c^	98.8	99.1	98.8	99.1	99.3	99.3
Medically impossible diagnoses^c^	96.5	97.1	97.4	97.8	99.1	99.1
Completeness	94.8	98.8	94.8	98.8	98.9	98.6
Length of cause list	100.0	100.0	100.0	100.0	100.0	100.0
Overall^d^	78.9	87.2	80.6	88.5	90.3	89.4

^a^Estimates for Mexico are from the Global Burden of Disease study for all age groups between 2013 and 2014

^b^Estimates for Yucatan are from the Global Burden of Disease study for all children under 5 years of age between 2015 and 2016

^c^Subtracted from one so that higher values are preferable to lower, as with other indicators

^d^Overall score is a product of the VSPI categories of garbage codes, age/sex unspecified, impossible diagnoses, and completeness

It is possible that the quality of death registrations varies depending on the place of occurrence of a death. To explore this, we recalculated the proportion of diagnoses that are medically impossible and the percentage of garbage codes, stratifying the deaths in three groups according to the place of occurrence: Ministry of Health hospitals, home, and other (mostly including hospitals from the Mexican Institute of Social Security-IMSS). For the proportion of garbage codes, the adjusted proportion was 93.1% for the Ministry of Health, 93.7% for other places, and 80.8% for deaths occurred at home. For the proportion of deaths with medically impossible diagnosis, these values were 96.7%, 98.3% and 94.2% respectively (Additional file 2). In both dimensions, the other category does better, followed by the MoH facilities and with lower values for deaths occurred at home, suggesting the quality of vital registration in these dimensions is lower for deaths occurred at home.

### Assessment of quality of cause of death information

At the individual level, concordance between underlying cause of death listed in the VR and the cause determined by the medical review varied substantially across causes. Overall, the mean chance-corrected concordance across causes for neonates was 6.9% (95% uncertainty interval (UI) −0.18, 31.4) and for children was 46.9% (95% UI 29.3, 63.7). This increased to 12.5% (95% UI −0.18, 49.5) and 60.7% (95% UI 39.1, 75.8) when considering any listed cause instead of only the primary underlying cause. At the population level, the CSMF accuracy was 35.3% (95% UI 34.2, 36.2) for neonates and 67.7% (95% UI 66.4, 68.0) for children. This increased to 38.0% (95% UI 36.7, 39.1) and 73.1% (95% UI 72.2, 73.8) when considering all listed diagnoses and not restricting to concordance for only the underlying cause of death. Table 3 shows the concordance between the VR and the cause derived from the medical record review.

**Table 3:** Mean chance-corrected concordance % (CCC) and cause-specific mortality fraction (CSMF) accuracy

Metric	Child (*n* = 130)		Neonate (*n* = 105)	
	Underlying diagnosis Mean (95% UI^a^)	All diagnoses Mean (95% UI)	Underlying diagnosis Mean (95% UI)	All diagnoses Mean (95% UI)
Individual				
CCC	46.9 (29.3, 63.7)	60.7 (39.1, 75.8)	6.9 (−0.18, 31.4)	12.5 (−0.18, 49.5)
Population				
CSMF Accuracy	67.2 (66.4, 68.0)	73.1 (72.2, 73.8)	35.3 (34.2, 36.2)	38.0 (36.7, 39.1)

^a^*UI* uncertainty interval

### Chance-corrected concordance (CCC) by cause of death

**Fig. 1:**
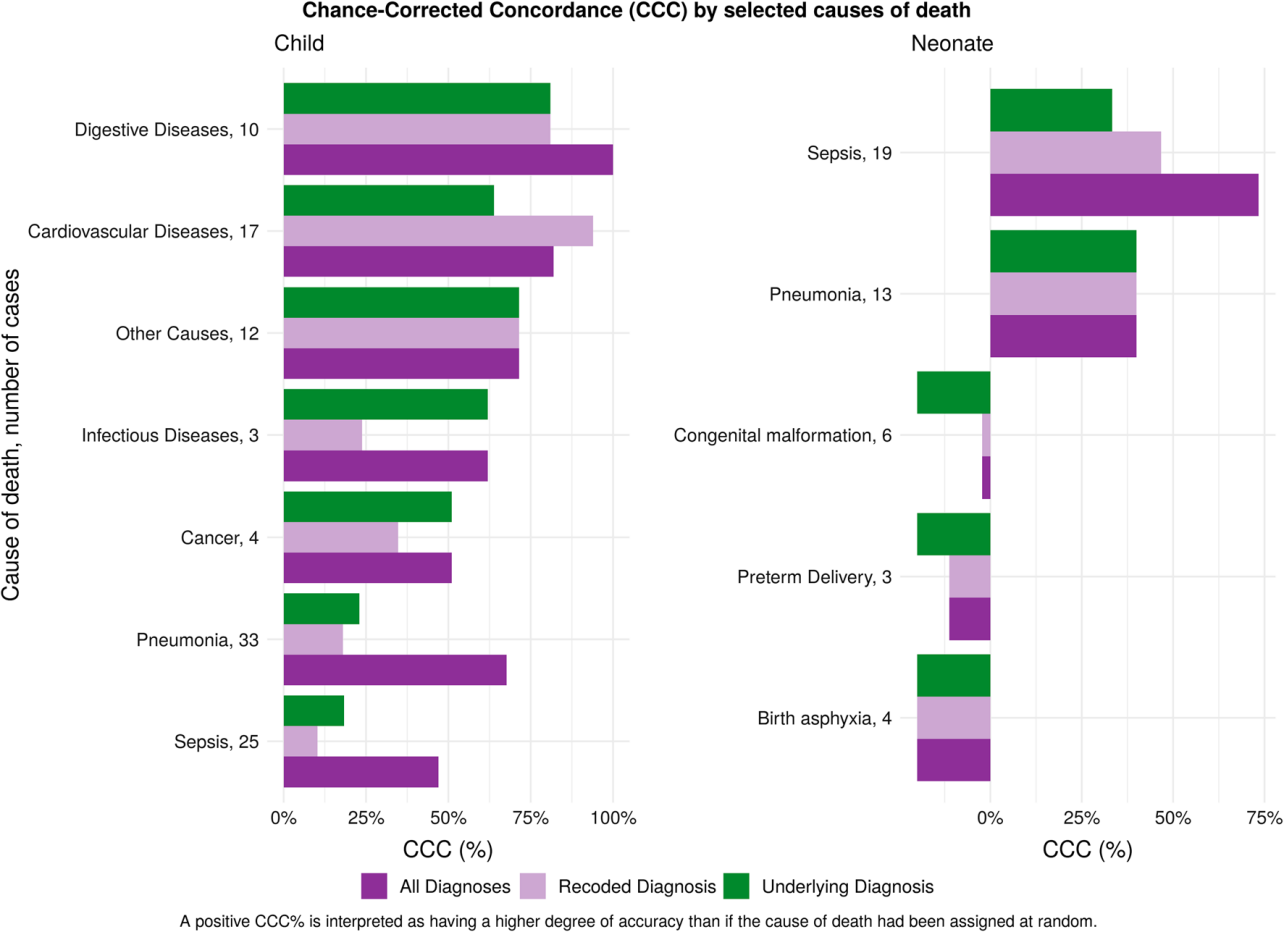
Chance-corrected concordance by cause using original primary diagnosis, recoded primary diagnosis, and all diagnoses

Figure 1 shows the CCC disaggregated by cause. There is substantial variation on the CCC by cause and each age group. For neonates, meningitis/sepsis had the highest concordance, followed by pneumonia. Preterm delivery, birth asphyxia, and congenital malformation all had negative chance-corrected concordance among neonates. For children, digestive diseases had the highest concordance for all diagnoses, followed by cardiovascular diseases. Diarrhea/dysentery had the lowest concordance for children.

### Cause-specific mortality fraction (CSMF)

**Fig. 2:**
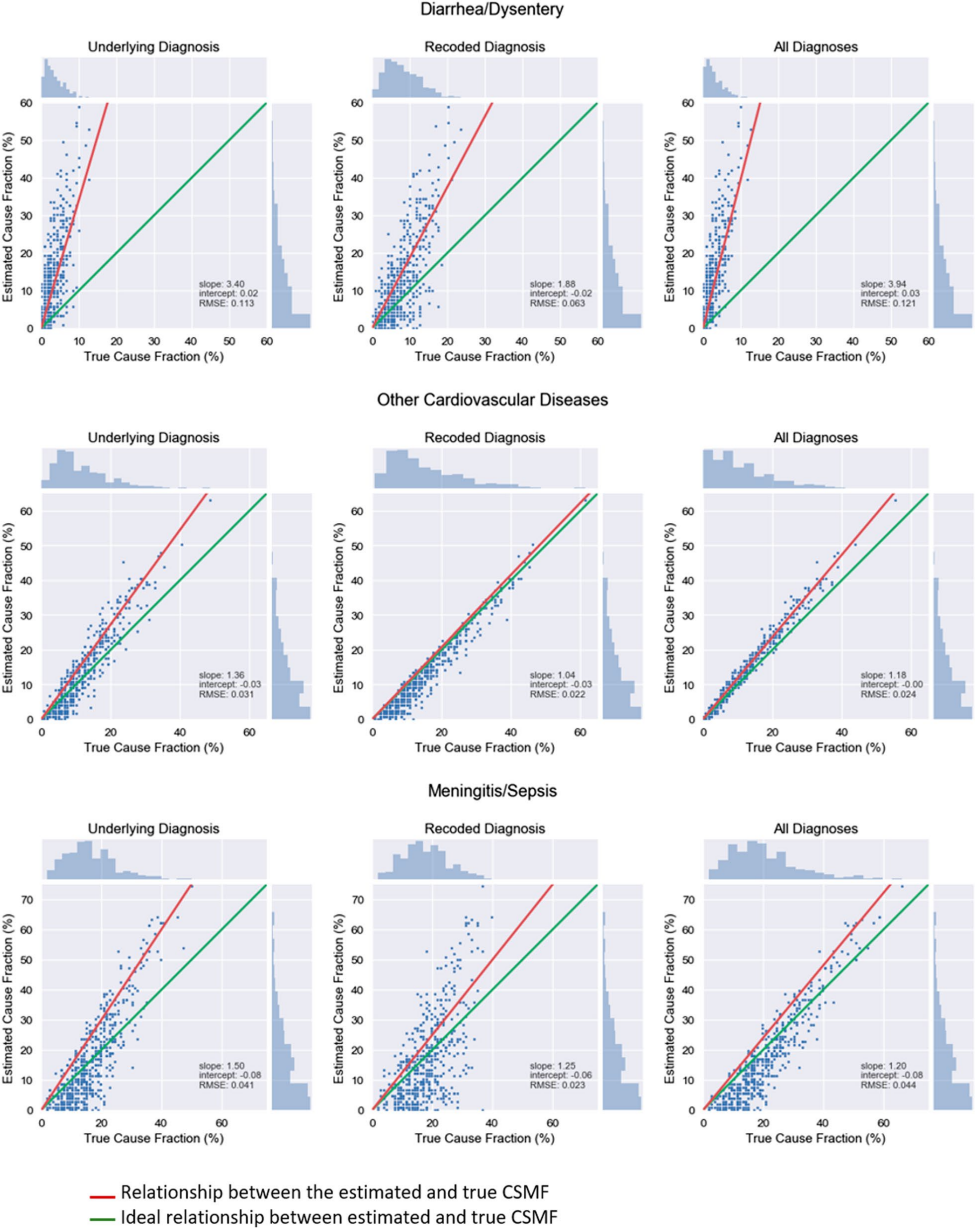
Regression fit results for CSMF resampling, diarrhea/dysentery and other cardiovascular diseases (children) and meningitis/sepsis (neonates). Green lines indicate an ideal relationship, whereas red lines indicate the estimated relationship between the true and estimated cause-specific mortality fraction (CSMF)

The CSMF accuracy estimate as determined by the resampling analysis varied across causes. Figure 2 shows the results for three causes selected to exemplify the heterogenous performance of the VR by cause: diarrhea/dysentery and cardiovascular diseases in children and meningitis/sepsis in neonates. The green line shows the ideal relationship in which the estimated CSMF perfectly matches true CSMF across the full range of possible true CSMFs. For most causes, the estimated CSMF overestimates the true CSMF, especially as the true fraction of the cause increases regardless of whether we analyzed the original underlying cause from the VR, the recoded underlying cause, or looked for concordance among all listed diagnoses. This can be seen by examining the slopes which are above 1, what is the case for diarrhea/dysentery. However, at a low true CSMF, most of the estimates for the three selected conditions are fairly accurate, as indicated by the intercept being close to zero. As is seen in these examples, using the recoded diagnoses produced estimates that were more precise, as indicated by the lower RMSE, and were less of an overestimate across a range of true CSMFs, as indicated by a slope closer to 1. A similar, though less consistent pattern is observed when examining all diagnoses compared to just the original underlying diagnosis.

### Recoding of causes of death

After recoding of the cause of death by an external team of coders, the underlying cause of death determined from the review and recoding of the death certificates disagreed with the original ICD-10 code listed in the vital registry for 317 (32.8%) cases. When both the original ICD-10 code and the recoded code were mapped to the GBD cause list, only 178 (19.0%) cases disagreed.

Chance-corrected concordance and the cause-specific mortality fraction were also calculated for the recoded values. For the individual level, the CCC mean for children was 44.6% (95% UI 27.2, 72.8), and for neonates, a mean was -1.39% (95% UI -25, 22.2). For the population level, the CSMF mean for children was 66.1% (95% UI 65.2, 66.8), and neonates had a mean of 29.3% (95% UI 28.0, 30.5).

## Discussion

The aim of this study was to assess the performance of the VR system in registering deaths of children under 5 in Yucatan, Mexico. The results of this study indicate that although the VR system has a good performance overall, with high scores in the dimensions used to measure its performance, there are still problems related to causes of death for children under 5, stemming mainly from the certification of causes of death. The estimation of the VSPI in Yucatan for children under 5 found levels of performance slightly lower than those calculated for Mexico for all age groups and for deaths of children under 5 in Yucatan using estimates from the GBD. The VSPI indicates that mortality registry is fairly complete, uses a detailed cause list, and contains a low number of cases with age or sex unspecified or with medically impossible diagnoses. However, the use of garbage codes as cause of death is the primary factor driving this index down, and the quality of death registration in terms of garbage codes and medically impossible diagnoses was lower for deaths occurring at home, suggesting that the analysis that we do about the cause of death on hospital deaths may be overestimating the quality of this process.

In our study, we were able to compare cause of death reporting from the VR to medical records as well as carefully recode causes from the review of the VR. While the extraction of information from medical records was done manually by trained personnel, it was captured in electronic methods which included an algorithm to classify the cases according to their gold standard certainty level. This constitutes an innovation that can have further application in studies analyzing electronic medical records. Comparing the cause of death from the VR against the ones derived from medical records allowed us to determine whether uncertainty or errors in the final statistics are due to incomplete diagnostics at the hospital or to mistakes when filling out death certificates. We used as metrics the chance-corrected concordance, which allowed us to estimate the agreement of the cause of death stated in the VR against the one derived from medical records, correcting for the possibility of getting it right just by chance. Negative chance-corrected concordance values occur when there are a small number of causes and the algorithm gets less than (1/n) fraction of the deaths correct for a given cause [19]. Investigating the concordance between the original and recoded VR using the GBD cause list provides a more sensible comparison. The GBD provides a smaller set of homogenous causes that are useful for public health reporting. In contrast, there are over 69,000 different ICD-10 codes. When viewed at this level of detail, minor discrepancies in coding may lead to drastic decreases in concordance. However, those minor disagreements may not lead to different actionable data. The fact that we did not find major differences in the cause of death as originally coded in the VR against the additional coding conducted in this study indicates that errors in the coding procedure may not generate substantial  errors in the ascertain of cause of death in vital statistics, and they may be rather due to problems in the certification process. In addition, finding a higher CCC when analyzing only the underlying cause of death or any cause of death stated in the VR against the one derived from medical records may indicate that the causes are mentioned in the death certificate, but there may be problems in the filling of the cause of death section that does not allow a right identification of the cause of death.

Interestingly, we found that the accuracy and concordance of cause of death information varied substantially across causes and age groups. The cause of death reported for neonates was generally less reliable than that of other children, getting a lower CCC overall. For neonates, deaths due to infectious diseases were more accurately reported than other causes of death like congenital malformations, preterm delivery and asphyxia, where the CCC is below 0, indicating a worse performance than if cases were assigned to causes at random. These results are consistent with a previous study conducted in Mexico, which also found lower CCCs in neonatal deaths than in children or adults [12]. In part, this may have to do with multiple comorbid neonatal conditions present for a single case and the difficulty associated with properly determining which is the true underlying cause. The opposite pattern was observed in children. In general, estimates of infectious diseases were less accurate than other causes. This could be due to the difficulty in determining the primary cause infectious agent, when either multiple infections are present or the patient only has a general profile of infection-related symptoms. In addition, documentation about care in medical charts was more limited in neonatal than pediatric deaths, which can be also due to more limited human resources in neonatal than pediatric wards (personal communication, Dr. Salvador Gómez Carro), which could affect the quality of the information about the cause of death in both the medical record and the death certificate.

During review of medical records, we uncovered cases which we were not able to link to records in the VR. In some cases, the reviewing physicians consulted hospital staff and determined that pieces of information were missing from the medical record, and the electronic submission form for reporting deaths did not accept incomplete records. Thus it is possible that these records were never submitted to the VR system for this reason. Another potential explanation for unmatched records could be deaths that were listed in the VR but were not able to be matched to the medical records because of incorrect or incomplete identifying information. This is likely given that we were only able to identify 77% of listed deaths at hospitals administered by the Ministry of Health. Both the unsubmitted records and the unlinkable records highlight areas that could be improved.

### Limitations

The study has some limitations that should be considered in the interpretation of results. In this study, we only examined deaths occurring at facilities operated by the Ministry of Health. This may affect our ability to generalize our conclusion to the entire state in three important ways. First, almost 20% of the deaths listed in the registry did not occur at a hospital. Given that records from hospitals were unable to make it into the registry because of administrative problems, it is even more likely that some community deaths are unreported and this is an underestimate. Furthermore, deaths that occur outside of hospitals may follow a different epidemiological profile. For example, fatal injuries are unrepresented in hospital deaths. Second, only about half of the hospital deaths listed a hospital administered by the Ministry of Health, and 73.8% of deaths with complete information in our study come from the main reference hospital in the State. People may select different hospitals for different medical needs, and the populations served by the public and private insurance schemes may have different profiles of risk factors and diseases [21]. All of these limitations would lead to potential bias in our quality assessment, overestimating the quality of death records in Yucatan, if we consider that deaths without medical certification or previous medical care are likely worse off in their ascertainment of cause of death than in-hospital deaths and that the causes of death may vary between deaths occurred at hospitals and those occurred at the communities.

## Conclusions

Cause-of-death information is recognized as one of the key statistics for developing well-informed health policy. The results of this study are consistent with previous studies done in Mexico and highlight the importance of strengthening mechanisms to improve the quality of cause of death certification at the hospital level. It is important to continually evaluate such systems in order to find steps where information is lost or distorted. This ensures that policymakers can use statistics that they know are complete and reliable.

Accurately assessing the quality and performance of VR systems is important for ensuring high-quality cause of death data suitable for use in making health policies. Using quantitative assessments to review VR systems can illuminate areas which are functioning well and areas that need improvement. Our review of the Yucatan VR systems shows that, for deaths under the age of 5, the State of Yucatan is performing very close to previously reported performance for the entire country, but that there is a large range of variation in the accuracy in the particular cause-specific estimates. This problem can be addressed through specific actions like training and supervision on death certification, which was conducted after discussion of these results with local stakeholders. Our review also highlights weak points in the operation of collecting and coding underlying causes of death. Reviews such as this are important for ensuring the quality and continued improvement of VR systems.

## Supplementary Information

**Additional file 1**. GC13 Adult Gold Standard Diagnoses.

**Additional file 2. Table 1:** Vital Statistics Performance Index (VSPI) scores by place of death for under-5 deaths in Yucatan, Mexico, 2015–2016.

## Data Availability

The datasets generated and analyzed during the current study are available in the Global Health Data Exchange repository, http://ghdx.healthdata.org/.

## Abbreviations

CCC: Chance-corrected concordance
CSMF: Cause-specific mortality fraction
GBD: Global burden of disease
ICD: International Classification of Diseases
MRR: Medical record review
PHMRC: Population Health Metrics Research Consortium
VA: Verbal autopsy
VR: Vital registry
VSPI: Vital Statistics Performance Index
