# Supplementary material for: Assessment of the quality of the vital registration system for under-5 mortality in Yucatan, Mexico
Source: Popul Health Metr. 2022 Feb 8;20:7. doi: 10.1186/s12963-022-00284-5 (PMC8822765; doi:10.1186/s12963-022-00284-5)
Supplement: Supplementary file 2 — Additional file 2. Table 1: Vital Statistics Performance Index (VSPI) scores by place of death for under-5 deaths in Yucatan, Mexico, 2015–2016. [file 12963_2022_284_MOESM2_ESM.docx]

Additional file 2

**Additional Table 1:** **Vital Statistics Performance Index (VSPI) scores by place of death for under-5 deaths in Yucatan, Mexico, 2015–2016**

|  | Original ICD Codes | | GBD Estimates | | Ministry of Health  (n = 431) | | Other place*  (n = 412) | | Home (n = 120) | |
| --- | --- | --- | --- | --- | --- | --- | --- | --- | --- | --- |
| *Dimension* | *Raw* | *Weighted* | *Mexico 2013* | *Yucatan 2014* | *Raw* | *Weighted* | *Raw* | *Weighted* | *Raw* | *Weighted* |
| Garbage codes^c^ | 87.4% | 91.8% | 92.8% | 92.1% | 89.4% | 93.1% | 90.4% | 93.7% | 71.3% | 80.8% |
| Medically impossible diagnoses^c^ | 96.5% | 97.1% | 99.1% | 99.1% | 96.1% | 96.7% | 98.1% | 98.3% | 92.5% | 94.2% |

*Other places include: IMSS, IMSS Oportunidades, ISSSTE, other public medical facility, private medical facility, other
